# An Automated System for Grading EEG Abnormality in Term Neonates with Hypoxic-Ischaemic Encephalopathy

**DOI:** 10.1007/s10439-012-0710-5

**Published:** 2012-12-04

**Authors:** N. J. Stevenson, I. Korotchikova, A. Temko, G. Lightbody, W. P. Marnane, G. B. Boylan

**Affiliations:** Neonatal Brain Research Group, University College Cork, Cork, Ireland

**Keywords:** Electroencephalography, EEG, Newborn, Neonate, Background, Wigner-Ville distribution, Multi-class linear discriminant classifier, Hypoxic-ischaemic encephalopathy, Automated EEG grading system

## Abstract

Automated analysis of the neonatal EEG has the potential to assist clinical decision making for neonates with hypoxic-ischaemic encephalopathy. This paper proposes a method of automatically grading the degree of abnormality in an hour long epoch of neonatal EEG. The automated grading system (AGS) was based on a multi-class linear classifier grading of short-term epochs of EEG which were converted into a long-term grading of EEG using a majority vote operation. The features used in the AGS were summary measurements of two sub-signals extracted from a quadratic time-frequency distribution: the amplitude modulation and instantaneous frequency. These sub-signals were based on a model of EEG as a multiplication of a coloured random process with a slowly varying pseudo-periodic waveform and may be related to macroscopic neurophysiological function. The 4 grade AGS had a classification accuracy of 83% compared to human annotation of the EEG (level of agreement, *κ* = 0.76). Features estimated on the developed sub-signals proved more effective at grading the EEG than measures based solely on the EEG and the incorporation of additional sub-grades based on EEG states into the AGS also improved performance.

## Introduction

Hypoxic-ischaemic encephalopathy (HIE) is a major cause of neonatal neurological morbidity in the developed world with an incidence of 2.5/1000 births.^12^,^16^ HIE results from a lack of oxygen and impairment to the blood supply in the neonatal brain around the time of birth and is an evolving injury with a secondary injury occurring hours after the initial HI insult.^2^,^16^ Recent advances in treatment of neonatal HIE, principally the use of therapeutic hypothermia (TH), have rekindled interest in methods of monitoring the cortical function of the newborn with HIE in the neonatal intensive care unit (NICU).^1^,^11^

The electroencephalogram (EEG) is capable of passively monitoring neonatal cortical function. It is portable, provides minimal disturbance to the neonate, has a high time resolution and is capable of long duration recording in excess of 48 h. The visual interpretation, or grading, of the background EEG (EEG without seizure or artefact) has been shown to be a useful tool when monitoring the recovery of cortical activity after a HI injury.^23^,^39^ In fact, the normalisation of the EEG after a HI injury, a process whereby the EEG recovers through EEG grades from no electrical activity at the time of injury through periods of bursting activity to more continuous EEG with discernible sleep states, has been shown to correlate with outcome at 2 years of age.^23^

The aim of visual interpretation is to grade a period of background EEG, typically an hour, as normal or abnormal and then to grade the degree of abnormality. This visual interpretation incorporates EEG characteristics such as amplitude, continuity, frequency content, symmetry, synchrony, sleep state cycling and clinical information such as the gestational age of the neonate, suspected diagnosis and any administered medications.^6^,^22^,^39^ There are several grading or classification systems based on slightly different interpretations of these EEG and clinical characteristics and most interpretations correlate with neonatal outcome.^44^

The major impediment to the widespread use of the EEG in the NICU is that its interpretation is difficult, time consuming, and must be undertaken by an experienced neonatal neurophysiologist. The fact that such expertise is not continuously available in many NICUs has resulted in the application of amplitude integrated EEG, a simplified version of EEG based on time and amplitude compression, and the development of automated analysis methods, particularly for seizure detection, to assist the non-expert’s assessment of cerebral activity.^10^,^32^ A real-time, automated method of analysing the EEG for abnormalities in cortical function would result in a more specific simplification of the EEG and has the potential to improve clinical management of neonates with HIE.

The concept of automatically grading the degree of abnormality in EEG has been developed for pediatric and adult populations.^7^,^33^ However, because of the marked changes seen in the EEG as the brain matures, these methods are not necessarily useful in a neonatal population.^22^,^25^ In neonatal populations, several methods have been proposed for the detection of important elements of an EEG grading system such as sleep stages and the burst suppression pattern.^21^,^29^ These methods would, nonetheless, require additional post-processing to generate a grading of neonatal EEG. Hathi *et al*.^15^ have developed a cerebral health index for babies (CHI/b), a form of automated grading system (AGS), based on quantitative assessment of the EEG. The details of this index, however, are undisclosed in the literature.

The aim of this study was to develop an automated system for grading the degree of abnormality in the EEG of term neonates with HIE. The use of automation *via* computation has the potential to simplify and accelerate the interpretation of the neonatal EEG for the NICU clinician who does not have expertise in neonatal electroencephalography. This paper constitutes a continuation of our previous preliminary study that analysed the applicability of several quantitative EEG (qEEG) features to differentiate between EEG grades in neonatal HIE.^20^

This paper is organised as follows: initially, the acquisition and grading of the neonatal EEG data are outlined. The AGS is then defined including a novel joint time-frequency signal model that was used to develop the EEG features that form the basis of the grading system. This results in a model-based AGS. Methods of optimising and analysing the AGS are then defined; from the optimisation of system parameters and feature selection to AGS assessment *via* cross-validation. The results of the optimisation and performance analysis, including an investigation of misclassified recordings, are then listed and discussed.

## Method

### Data Acquisition

Continuous video-EEG data were recorded using the NicoletOne EEG system with an 8-channel bipolar montage (Carefusion Neurocare, Wisconsin, USA). This study had full approval from the Clinical Ethics Committee of the Cork Teaching Hospitals and written informed parental consent was obtained for all infants studied.

The patterns seen in the EEG can vary greatly with conceptional age and condition, therefore, only the EEGs of term neonates with HIE were used. These neonates were not treated with therapeutic hypothermia as they were recorded before 2006 when the treatment was adopted by our NICU. The EEG was commenced, typically, within 12 h of birth and continued for 24–72 h in order to monitor the progression of the developing encephalopathy.^22^,^25^ The EEG was initially filtered in order to avoid aliasing and DC drift and then sampled at 256 Hz. The data were downsampled to 64 Hz for further processing as a computational saving and because the EEG activity of interest in neonates is negligible in frequencies over 32 Hz.

**Figure 1:**
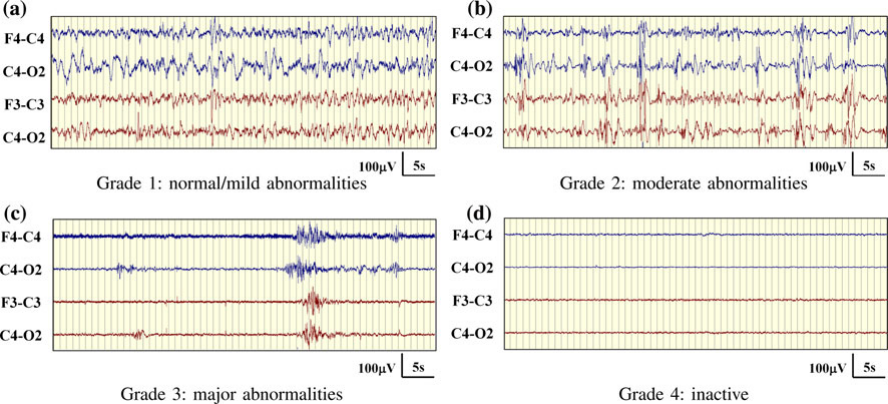
Representative EEG epochs from ideal EEG grades

Approximately one hour of EEG data, free of seizures and major movement artefacts (amplitude higher than 250 μV lasting for more than 3 sec), was selected from the recordings of 54 newborns. The segments of EEG were selected to ensure a relatively constant EEG grade was present within the hour. All other minor artefacts were annotated in the recording. EEG epochs containing minor artefacts were excluded during training but included when in testing on unseen data (validation). Artefact free epochs were used in training as a final AGS would be expected to include an artefact rejection system. All one hour EEG files were independently graded by two experienced neonatal EEGers (IK and GBB). In those cases when two different grades were assigned to the same EEG file, the EEGs were subsequently reviewed, discussed by the EEGers and consensus on the EEG grade was reached. The inter-observer agreement between both EEGers was high (the inter-observer agreement as measured using Cohen’s *κ* statistic was 0.868^8^). This corresponds to a disagreement in 4/54 EEG recordings. Example EEGs from each grade are shown in Fig. 1.

The visual classification system used to assess the EEG was adapted from the system used by Murray *et al*.^23^ and was specifically developed for term neonates with HIE. This classification system is summarised in Table 1. The neonatal EEG was assigned one of 4 grades corresponding to normal/mild abnormalities (grade 1), moderate abnormalities (grade 2), major abnormalities (grade 3) and inactive (grade 4). Periods where the EEG signal characteristics significantly change were also annotated. These periods correspond to sleep states in normal EEG, but are effectively disrupted sleep states in abnormal EEG. This results in the additional annotation of S1 (normal or disrupted quiet sleep and indeterminate sleep) and S2 (normal or disrupted active sleep and awake). These supplementary grades are only seen in grades 1 and 2, as cycling between EEG states disappears in higher grades of abnormality.^19^

The database consisted of 62 h of 8-channel EEG recording and consisted of 22 neonates with grade 1 EEG, 14 neonates with grade 2 EEG, 12 neonates with grade 3 EEG and 6 neonates with grade 4 EEG. The median recording duration was 65 min (interquartile range IQR: 62–67 min) and the median duration of minor artefact on the recordings was 30 min (IQR 18–46 min). Additional demographics and the inclusion criteria of the cohort can be found in Korotchikova *et al*.^20^

### Automated Grading System

**Figure 2:**
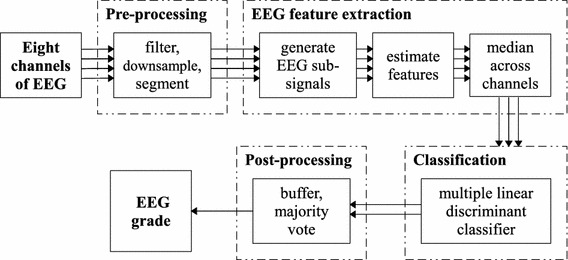
The automated grading of one hour of neonatal EEG

The proposed grading system used a simple feature extraction and classification paradigm with pre-processing and post-processing stages. The pre-processing stage involved an initial filtering step (a high pass FIR linear phase filter with an order of 1858—a transition width of 0.5 Hz), downsampling (256–64 Hz) and segmentation of the EEG into short analysis epochs with a 50% overlap. The feature extraction stage involved the estimation of a series of features (summary statistics with respect to time) from two EEG signal derivatives estimated on each epoch of EEG. These EEG signal derivatives, the amplitude modulation (AM) and the instantaneous frequency (IF), were designed to match important aspects of the visual interpretation of the neurophysiologist. As HIE is assumed to be a global injury, the information between EEG electrodes was combined by taking the median across 8-channels of the bipolar EEG montage.^21^ The features were passed to a multi-class linear discriminant classifier (MLDC) to determine an EEG grade. At this stage, an expanded grading was used (1S1, 1S2, 2S1, 2S2, 3, 4) in order to incorporate the temporal changes of EEG state seen in grades 1 and 2. The post-processing stage involved buffering an hours worth of decisions, combining EEG states with similar grades (reducing the grades from 6 to 4), followed by a majority vote operation. This operation selects the most common (maximum likelihood) decision within the hour. The proposed AGS is shown in Fig. 2.

The features used in the AGS were based on a model of the neonatal EEG background signal. The EEG signal is a measurement of the averaged macroscopic electrical field, generated by the firing of cortical neurons, after it has passed through the cerebrospinal fluid, skull, and scalp.^25^,^27^,^43^ The neonatal background EEG signal exhibits both stochastic and chaotic characteristics that change in amplitude and frequency content over time.^26^,^36^,^37^ The changes with time (nonstationarities) can occur over durations ranging from seconds to hours. The neonatal EEG is in addition recorded at several locations on the scalp and as such, exhibits spatial variability.

The neonatal EEG background has been modelled as a coloured noise process.^30^,^36^,^37^ This results in a model of neonatal EEG background as,

1 $$ \mathrm{EEG}(t) = X(t) = T_{\mathrm{filter}}\{ Y(t) \} $$

where the transformation $T_{\mathrm{filter}}\{\cdot\}$ can be a linear or nonlinear filter and *Y*(*t*) is white Gaussian noise. The frequency response of the simulating filter tends to have a power law response of 1/*f* ^α^.^30^,^36^,^37^ This power law varies over time to account for the nonstationary behaviour of the EEG. A major deficiency of this model is that it does not take into account the AM seen in EEG patterns of the term neonate such as tracé alternant and burst-suppression which are associated with QS and EEG grades 2 and 3, respectively.^44^ Including AM into this signal model results in a model of EEG,

2 $$ \mathrm{EEG}(t) = a_m(t) X(t) $$

where *a*_*m*_(*t*) is the AM component. As (2) is a dual or canonical function, there are an infinite number of choices of *a*_*m*_(*t*) and *X*(*t*) that can generate the EEG signal, $\mathrm{EEG}(t).$ 3 If the frequency content of *a*_*m*_(*t*) is constrained to be much less than *X*(*t*), then information on the underlying signal transformation $T_{\mathrm{filter}}\{\cdot\}$ can be estimated from the frequency domain representation of *X*(*t*) (*via* estimates of the IF). This leads to an the interpretation of *a*_*m*_(*t*) and $T_{\mathrm{filter}}\{\cdot\}$ which aligns with the visual interpretation of the EEG made by the neurophysiologist of amplitude and frequency content, respectively. These components may also relate to neurophysiological function as the AM and IF may represent different aspects of the influence of cortico-cortical and thalamo-cortical connections on EEG generation.^35^,^40^

It is assumed that information within the time-varying amplitude and frequency of EEG signal can be used to automatically grade the degree of abnormality in the EEG of term neonates with HIE.

This signal model of EEG can, therefore, be defined in the joint energy-time-frequency domain as,

3 $$ \left|\mathrm{EEG}(t,f)\right|^2 = \left|\frac{a_m(t)}{f^{\alpha(t)}}\right|^2. $$

The AM and IF sub-signals can be estimated from the EEG signal using the analytic associate of the signal or quadratic time-frequency distributions (QTFD).^3^ In this case, QTFDs are used to form an estimate of (3). The general class of TFDs, the quadratic class, can be formed from the Wigner-Ville distribution (WVD) and is defined as,^3^

4 $$ \rho_\gamma(t,f) = W_z(t,f) \mathop{\ast \ast}\limits_{(t,f)}\gamma(t,f) $$

where ** is a two-dimensional convolution operation, *γ*(*t*, *f*) is the filter that characterises different TFDs within the quadratic class, such as the spectrogram, separable kernel, or Choi-Williams distribution, and *W*_*z*_(*t*, *f*) is the WVD which is defined as,^3^

5 $$W_z(t,f) = \int\limits_{-\infty}^{\infty}z(t+\frac{\tau}2){z}^\star(t-\frac{\tau}2) e^{-j2\pi f \tau} \;d\tau $$

where, *z*(*t*) is the analytic associate of the real signal under analysis, that is, $z(t) = EEG(t)+j\mathcal{H}\{EEG(t)\}$ where $\mathcal{H}$ is the Hilbert transform, $z^\star(t)$ denotes the complex conjugate of *z*(*t*), *f* is frequency, *t* is time and τ is time lag.

The AM and IF (*f*_*i*_(*t*)) can be approximated from a QTFD,

6 $$ a_m(t) = \left(\int\limits_{-\infty}^{\infty} \rho_\gamma(t,f) \; df\right)^{0.5} $$

7 $$ f_i(t) = \frac{\int_{-\infty}^{\infty} f \rho_\gamma(t,f) \; df}{\int_{-\infty}^{\infty} \rho_\gamma(t,f)\;df}.$$

A smoothed QTFD (*γ*(*t*, *f*) is a two-dimensional Hamming window, bandwidth 1 Hz, duration 1 s) was used to estimate the AM and IF sub-signals. A smoothed QTFD was used as it results in a non-negative representation, as the bandwidth-duration (BT) product of the smoothing window is greater than or equal to one.^17^ This results in non-negative AM and IF sub-signals (negative energy and negative frequency do not have physical correlates).

Assuming a slowly time-varying transformation, $T_{\mathrm{filter}}\{\cdot\} = 1/f^{\alpha(t)},$ and a bandlimited process defined by lower (*f*_1_) and upper (*f*_2_) cutoff frequencies, then the relationship between the IF and α(*t*) can be estimated by substituting (3) in (7),

8 $$ f_i(t) = \frac{\left(f_2^{2-2\alpha(t)} - f_1^{2-2\alpha(t)} \right)\left(1-2\alpha(t) \right)}{\left(f_2^{1-2\alpha(t)} - f_1^{1-2\alpha(t)} \right)\left(2-2\alpha(t) \right)} \quad {\text{where}} \;\; \alpha(t) > 1, $$

**Figure 3:**
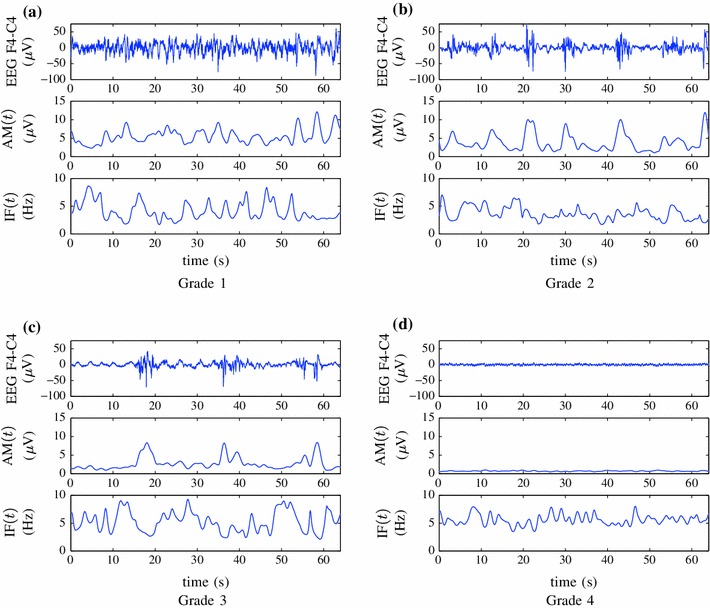
AM and IF of ideal EEG epochs

*f*_2_ is the Nyquist rate, which in this case is 32 Hz, while *f*_1_ will be optimally selected between 0.5 and 3 Hz in order to eliminate low frequency artefact from the EEG recording. Examples of the AM and IF for a 64 s epoch of EEG for each grade is shown in Fig. 3.

Several features were calculated on the AM and IF sub-signals. These features include the mean, standard deviation, skewness, and kurtosis and were estimated with respect to time. Each epoch of EEG was, therefore, represented by 8 values estimated from the AM and IF. It must be noted that summary statistics of the IF will not necessarily correlate with higher order moments calculated on the frequency marginal of the QTFD (an estimate of the power spectral density).

The qEEG features of relative delta power, EEG skewness and EEG kurtosis were incorporated into this feature set based on previous work into EEG classification.^20^ Additional measures of covariance between the AM and FM, symmetry (revised brain symmetry index,^41^) and synchrony (maximum and argument of the maximum of the correlation function estimated on *a*_*m*_(*t*)) and interburst interval (IBI) estimated from the algorithm of Palmu *et al*.^28^ were also included. The IBI estimation algorithm was implemented with no modification as preliminary analysis showed high correlation between actual and estimated IBI. The initial feature set that was used in training is summarised in Table 2. All features were normalised using a Box-Cox transformation during training to ensure similar shape, mean and standard deviation of the distribution of each feature.^4^

**Table 1:** Classification of the visual interpretation of EEG background activity in HIE

EEG grade	Cerebral function	Description
1	Normal/mild abnormalities	Continuous background pattern with normal/slightly abnormal activity (mild asymmetries, mild voltage depression [30–50 μV]), presence of SWC that might be poorly defined
2	Moderate abnormalities	Discontinuous activity with IBI of ≤10 s, disrupted SWC, clear asymmetry or asynchrony
3	Major abnormalities	IBI of 10–60 s, lack of variability, severe attenuation of background patterns [<30 μV], absence of SWC
4	Inactive	Background activity <10 μV, or severe discontinuity with IBI ≥ 60 s

Increasing grades are associated with reduced cerebral function

IBI—interburst interval, SWC—sleep wake cycle

The decision making within the AGS is performed by a MLDC.^9^ This classifier is designed to find a linear projection of the features that maximise the Fisher discriminant. This discriminant is a function of the distance between the means of the classes and the covariance of the data within the classes and is defined, for a two class problem, as,

9 $$ J({\mathbf{w}})=\frac{|\tilde{m}_1-\tilde{m}_2|^2}{s_1^2+s_2^2} $$

10 $$ \tilde{m}_i = 1/n_i \sum_{y \in {\mathcal{Y}}_i} y $$

11 $$ \tilde{s}_i^2 = 1/n_i \sum_{y \in {\mathcal{Y}}_i} (y-\tilde{m_i})^2 $$

where $y = \mathbf{w^tx},$ $\mathbf{x}$ contains samples from each class (each sample is typically a vector of features), $\mathcal{Y}_i$ represents a subset/class of the linear combination of the components of $\mathbf{x}$ (*i* = [1, 2]), *n*_*i*_ is the number of samples in each class, $\mathbf{t}$ is the transpose operator, $\tilde{m}_i$ and $\tilde{s}_i$ are the mean and scatter of the samples from each class projected onto line $\mathbf{w}.$ 9 It essentially measures the Mahalanobis distance of a pattern towards the class centers. In order to classify *C* classes (*C* > 2), a pairwise classification based on each pair of classes is used (resulting in *C*(*C* − 1)/2 classifiers in total). The decisions of the pairwise classifiers are then combined to produce a final classification.

This classifier was chosen as it is computationally fast which is useful when a large number of training iterations are to be undertaken.

### AGS Evaluation

The performance of the AGS was evaluated with a leave-one-subject-out (LOSO) cross-validation. A LOSO cross-validation results in a near unbiased estimate of the true generalisation error; this means that the performance of the AGS estimated with a LOSO cross-validation will be most similar to the performance of the AGS applied to an infinitely large unseen set of EEG recordings once trained on all available data.^42^

A training set was formed by leaving out a single subject from the full database resulting in a 53:1 split of subjects between training and testing data. A randomly selected subset of the training set (7 min per 53 neonates) was used to optimise the pre-processing parameters, perform feature selection and train the MLDC. The performance of the trained AGS was then estimated on the full EEG recording of the left out subject and the process was repeated until all subjects had been left out of a training set. The results were then averaged across 54 iterations of the LOSO cross-validation.

The performance metric used in testing, and the cost function to be maximised during training was Cohen’s *κ* statistic estimated on the post-processed output (majority vote). During training, if more than one parameter set corresponded to the maximum, the maximum *κ* on the short-term grading results was used to break the deadlock. Cohen’s *κ* statistic is a measure of the agreement between the AGS and the EEGer and is defined as,^8^

$$ \begin{aligned} \kappa &= \frac{p_a - p_e}{1 - p_e} \\ p_a &= \sum^M_{i=1} p_{ii}\\ p_e &= \sum^M_{k=1} \left(\sum^M_{i=1} p_{ik} \sum^M_{j=1} p_{kj}\right) \end{aligned} $$

where *p*_*a*_ is the grading accuracy, *p*_*ij*_ is the proportion of neonates graded as *i* by the AGS and *j* by the EEGer and *M* = 4 is the number of EEG grades.

The AGS requires the selection of several parameters and definition of the optimal feature set before implementation. The pre-processing parameters, epoch length and cutoff frequency of the high-pass filter were greedy-searched in the range [8, 64] s for epoch length and [0.5, 3] Hz for the cutoff frequency. This was performed using a nested LOSO cross-validation applied to the training set of 53 subjects. An MLDC classifier, as a wrapper, was trained on 52 training subjects and evaluated on the remaining training subject. The cost function (*κ*) was estimated by averaging across 53, 52:1 split of training subjects. The parameters with the highest classification accuracy were selected.

**Figure 4:**
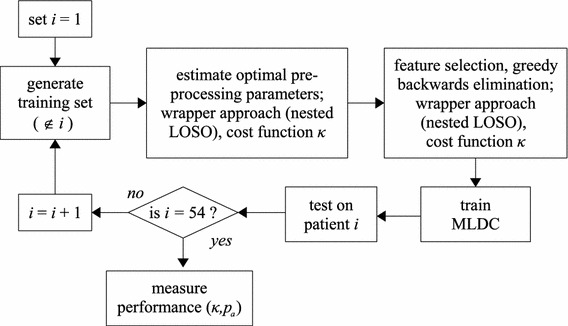
Performance assessment of the AGS using a LOSO cross-validation

Similarly, in the same nested LOSO loop, a greedy, backwards elimination feature selection procedure was implemented to generate an optimal feature set.^13^,^18^ Using an MLDC classifier as a wrapper, the cost function (*κ*) was estimated as per the pre-processing optimisation procedure. This way, the model selection routine (the nested LOSO) is completely independent of the performance assessment routine (the main LOSO) and the testing subject was not seen or used at any time for any system parameter tuning. The evaluation of AGS performance using a LOSO cross-validation is depicted in Fig. 4.

## Results

The training component of the LOSO cross-validation results in 54 different, optimal combinations of pre-processing parameters, selected feature sets and MLDCs. The optimal (maximum likelihood) pre-processing parameters and the search range are given in Table 3. The epoch length was limited to 64 s as several EEGs did not have artefact free training epochs of longer duration.

**Table 2:** The features trialled for the AGS

Feature type	EEG feature
AM	Mean, standard deviation, skewness, kurtosis
IF	Mean, standard deviation, skewness, kurtosis
AM/IF	Covariance
Additional qEEG	Relative delta power, skewness of the EEG, kurtosis of the EEG, IBI
Inter-hemispheric	Symmetry, synchrony

The most commonly selected number of features was 8 (15/54) and the most commonly selected features (selected >27 times) were mean AM (54/54), standard deviation AM (54/54), mean IF (40/54), kurtosis IF (36/54), kurtosis AM (35/54), and skewness IF (32/54).

The median *κ* across all training iterations of the LOSO was 0.87 (IQR: 0.84–0.89). The median *p*_*a*_ across all training iterations of the LOSO was 91% (IQR: 89–93%).

The performance of the AGS with the optimal feature set on the testing iterations of the LOSO cross-validation are shown in Table 4. The results were compared to a MLDC based on a feature set consisting of five qEEG features (mean AM, standard deviation of the AM plus the qEEG features listed in Table 1) that have previously been shown to differentiate between EEG grades.^20^ This was implemented in order to assess any improvements due to new features based on the AM/IF EEG signal model. The AGS was also implemented with a 4 grade MLDC in order to assess any improvements due to the incorporation of sub-grades relating to sleep state within grades 1 and 2.

**Table 3:** The optimal pre-processing/feature extraction parameters and search range

Parameter	Value	Search range	Selected
Pre-processing HPF cutoff	2.5 Hz	[0.5, 3] Hz	33/54
Pre-processing epoch length	64 s	[8, 64] s	54/54

**Table 4:** LOSO performance of the AGS

	*p*_*a*_ (%)	*κ*
qEEG feature set (4 states)	72.2	0.602
qEEG feature set (6 states)	75.9	0.659
modified feature set (4 states)	77.8	0.681
modified feature set (6 states)	83.3	0.762

**Table 5:** The agreement between the AGS and the EEGer

	AGS output				Total
	1	2	3	4	
EEGer output					
1	20	2	0	0	22
2	3	10	1	0	14
3	0	3	9	0	12
4	0	0	0	6	6
Total	23	15	10	6	54

The results are with an AGS based on 6 initial states (reduced to 4 in post-processing) and the modified feature set

**Figure 5:**
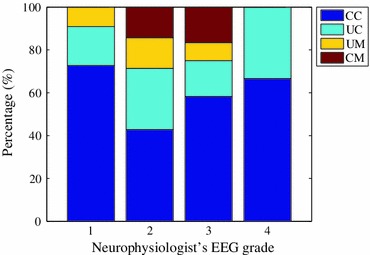
The distribution of certainty across AGS decisions. CC is certainty in correct classification, UC is uncertainty in correct classification, CM is certainty in misclassification and UM is uncertainty in misclassification

The confusion matrix outlining the agreement between the AGS output and the EEGer when grading an hour of neonatal EEG is outlined in Table 5. In total, the EEG recordings of 45 neonates were correctly classified with 9 EEG recordings misclassified. In order to assess the level of certainty of decision, a modification to the post-processing stage was introduced that requires at least a two-thirds majority for a certain decision. Any decisions below this level of certainty were deemed uncertain. In this case, 33 neonates were classified correctly with certainty, 12 neonates were classified correctly with uncertainty, 4 neonates were misclassified with certainty and 5 neonates were misclassified with uncertainty. The distribution of certainty across AGS decisions is shown in Fig. 5.

Nine recordings were misclassified, of these, 4 were misclassified with certainty. One EEG recording had been incorrectly assigned due to human error by both EEGers, however the AGS decision was deemed accurate. Two EEG recordings were misclassified due to the presence of abnormalities such as asymmetry, asynchrony and runs of sharp waves. These abnormal patterns were more prevalent in a decision critical EEG state (S1 or S2) which, in addition, tended to be of shorter relative duration than the alternate EEG state. The final recording was assigned a grade 2 by the AGS and a grade 3 by the human rater, as the burst-suppression pattern (the IBI was <10 s) was persistent, asymmetric, asynchronous and lacked variability across the hour of EEG recording. It is also interesting to note that one of the four certain misclassifications corresponded with a disagreement between EEGers. The remaining misclassifications were uncertain decisions and in three of these the misclassifications were caused by long periods of artefactual EEG which were incorrectly graded by the AGS. Artefact free periods in these three recordings were correctly graded.

**Figure 6:**
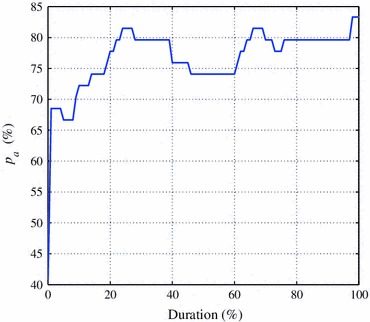
The affect of recording duration under analysis on the performance of the post-processing stage

The effect on EEG recording duration on the AGS decision was then estimated (see Fig. 6). An acceptable level of accuracy was achieved using approximately 25% (15 min) of the EEG recording.

The median error rate per epoch was 20% (IQR: 3–37%) in artefact free data compared to 29% (IQR: 8–56%) in data contaminated with artefact.

## Discussion

This paper extends the initial exploratory data analysis demonstrating the association between a combination of several qEEG features and grades of EEG abnormality assigned by experienced EEGers in term neonates with HIE.^20^ The extension formulates this work, within a pattern recognition paradigm, into an automated EEG grading system. Several qEEG features involving amplitude and frequency content are replaced with an alternate definition based on a novel EEG signal model. The extraction of these features, the number of features used in the AGS and the parameters related to the pre-processing stage were then all optimised on a small set of neonatal EEG. The MLDC, the core of the AGS, was then trained and tested within a LOSO cross-validation which permits an estimate of the AGS performance on unseen neonatal EEG recordings.

The use of model-based features improves the grading performance of the AGS compared to our previous exploratory data analysis.^20^ While a high level of performance was achieved in our previous work (91%), these results were optimistic as they were estimated on training data only. When the qEEG features identified in this work were applied to unseen data, *via* a LOSO cross validation, a significant drop in grading performance was noted (72%). A similar drop in performance between training and testing data was not, however, seen in the proposed AGS.

Normal reassuring EEG in neonates with HIE has normal amplitude, is continuous and contains discernible sleep states. Increasing abnormality of the EEG results from an increasing level of cortical deactivation that reveals bursting EEG patterns (grades 2 and 3) with increasing IBI, where the influence of sub-cortical activity is more pronounced, decaying to an eventual absence of cortical activity (grade 4).^31^,^34^,^40^ The AM component of the EEG best represents increases in discontinuity or bursting EEG patterns and the overall EEG amplitude. Supplemental information from the IF component of the EEG represents more subtle changes in the frequency content of cortical function and can be used to discriminate between EEG grades 1 and 2.

The parameters used in pre-processing affect the performance of the AGS. The optimal epoch length used was 64 s, implying stationarity among features over this duration. It may also be optimal as 60 s is used in the visual interpretation to differentiate between the IBI in grades 3 and 4. This is different from methods of automated seizure detection which use much shorter analysis epochs as the definition of a minimum seizure duration is 10 s. A longer analysis epoch, however, is used in methods of sleep state and burst suppression detection.^21^,^29^,^38^ It must be noted that epochs longer than 64 s could not be tested as several neonates did not have artefact free EEG periods of such duration. The optimal high pass frequency cutoff was 2.5 Hz. This was a surprising selection as there is significant EEG activity present in the delta band (0.5–4 Hz) and even lower frequencies.^5^,^40^ This suggests that there was discriminatory information in higher EEG frequencies and/or there was low frequency EEG activity that interfered with classification.

The discriminatory information extracted from the EEG was focused on the first two central moments of the AM. It is well known that the average amplitude and discontinuity (standard deviation of the AM) are a particularly useful aspect of the visual classification of neonatal EEG.^20^,^39^,^44^ This information was not enough to provide optimal grading accuracy and needed to be complemented with information from the central moments of the IF, suggesting that the distribution of the IF in time was associated with EEG grade. No residual qEEG features were predominant in the feature selection process. This implies that summary statistics of the IF provide more useful information to the classifier than other measures of frequency content, such as relative delta power and spectral edge frequency.

It is apparent that the visual interpretation guidelines in Table 1 for grading the abnormality of the EEG are not exhaustive. There is still disagreement by neurophysiologists and neurologists to specific aspects of a grading system regarding the interpretation of abnormal EEG patterns such as left-right and anterior-posterior hemispheric symmetry and synchrony, runs of spikes, sharp waves and epileptiform discharges.^44^ There are also potential modifications to the visual grading of EEG that may result due to the application of TH to neonates with HIE.^14^,^24^ It is expected that this would result in a dual mode AGS, where knowledge of the temperature of the neonate would be required, to correctly grade the AGS. The dual mode aspect of the AGS is required as while the primary application of the AGS would be to monitor sick neonates undergoing TH, it can also be used within 6 h of birth to provide additional information on cortical function to the clinician who is deciding whether or not to administer TH.

The use of a small number of features summarised over a relatively short period of time implies that a relatively limited amount of information was required to accurately grade the EEG. This suggests that there may be potential to improve the resolution of the EEG classification (increase the number of grades). In contrast, the analysis of misclassification implied that some abnormalities were more prominent during a particular EEG state. Although the duration of the analysis window may be reduced with no loss of grading accuracy, the total length of the EEG recording may need to be increased to ensure the decision-critical EEG state is recorded. This can be taken into account in the post-processing stage or by incorporating methods for automatically segmenting the neonatal EEG into disrupted sleep states.^29^ Additionally, the spatial resolution of the AGS can be improved (removing the assumption of a global injury) from left-right and anterior-posterior spatial relationships down to areas of the cortex covered by single electrodes.

The presence of artefact resulted in a 9% increase in the epoch error rate. The influence of artefact was, however, not uniformly distributed and contributed to an incorrect decision in 33% of misclassified EEG recordings. This suggests that while the AGS could overcome artefact on most EEG recordings by post-processing an hours worth of decisions, additional work is still required to improve the robustness of the AGS when more dominant contamination of the neonatal EEG occurs. One possible avenue of investigation is to include an artefact grade and artefact specific features into the AGS which can then be analysed by the post-processing stage, tempering decisions on artefact laden EEG recordings. The robustness of the AGS must be further developed before validating on a independent set of EEG recordings.

The incorporation of methods to detect asymmetry, asynchrony and runs of sharp waves also have the potential to improve the AGS even though they were routinely discarded during feature selection. The presence of these waveforms in the EEG recording results in a downgrade of the estimated AGS grade. The inclusion of these EEG phenomenon into the current AGS is not trivial as they are difficult to detect and appear intermittently (they cannot be post-processed with a majority vote). We intend to continue development of the AGS until it can be applied confidently in the NICU. The resultant AGS has the potential to provide important information on cortical function to the clinician who has limited expertise in the visual interpretation of the complex neonatal EEG signal.

## Conclusion

A method for automatically grading the degree of EEG abnormality in neonates with HIE was proposed. Modelling of the background EEG signal resulted in the division of short epochs into AM and IF sub-signals. These sub-signals were extracted from a positive QTFD. Summary measures of these sub-signals were then assigned to one of six grades of short-term abnormality by a MLDC which was then post-processed into one of four long-term EEG grades. This method was capable of correctly grading 83% of EEG recordings from 54 neonates. Future work includes incorporating measures of sleep state cycling into decision making, applying advanced methods of classification, alteration of the post-processing stage to include intermittent features and improving the robustness of the system to contamination by non-cortical sources.
